# Antibiotic and growth factor-loaded hydrogels for infected bone defect repair: mechanisms of spatiotemporal delivery and cellular microenvironment regulation

**DOI:** 10.3389/fcell.2026.1996917

**Published:** 2026-10-27

**Authors:** Lin Chen, Xulin Zhang, Lan Luo, Jiaping Lan, Xin Deng, Yang Wen, Lei Li

**Affiliations:** 1 Traumatic Orthopedics, Suining Central Hospital, Suining, Sichuan, China; 2 Hand Micrographic Surgery, Suining Central Hospital, Suining, Sichuan, China; 3 Gastroenterology Department, Suining Central Hospital, Suining, Sichuan, China

**Keywords:** infected bone defect, hydrogel, antibiotic, growth factor, synergistic mechanism, sequential release

## Abstract

The treatment of infected bone defects (IBDs) requires simultaneous infection control and bone regeneration. However, traditional staged treatment protocols have several limitations. These include extended treatment durations, multiple surgical procedures, and low repair efficiency. Bifunctional hydrogels loaded with antibiotics and growth factors (GFs) offer a crucial material platform for integrated treatment strategies. These hydrogels possess excellent biocompatibility, malleability, and the capacity for spatiotemporal drug delivery. This review summarizes the design principles of antibiotic- and GF-loaded hydrogels, with particular attention to sequential and synergistic release strategies and their roles in infection control, immune regulation, vascularization, and osteogenesis. Current *in vitro* and *in vivo* evaluation approaches are also discussed, together with remaining challenges in drug stability, release precision, long-term biosafety, and clinical translation. This review may provide a basis for the rational design of multifunctional hydrogel systems and their further development for infected bone defect repair.

## 1 Introduction

Infected bone defects (IBDs) present dual pathological characteristics of persistent infection and impaired bone regeneration. They remain one of the major challenges in the field of orthopedic repair. Currently, clinical practice mostly adopts a staged treatment strategy (Cui et al., 2026). This involves a primary thorough debridement combined with antibiotics to control infection. Subsequently, a secondary bone grafting procedure is performed to repair the defect. Although this approach can control infection to some extent, it is associated with several issues (Zhao et al., 2025). These include multiple surgeries, extended treatment durations, high costs, and difficulties in determining the optimal timing for repair. Therefore, researchers have devoted extensive attention to developing new bioactive materials. These materials aim to integrate local anti-infection properties and bone regeneration induction within a single system (Tian et al., 2024). Hydrogels are considered crucial carriers for constructing such integrated treatment platforms (Chen et al., 2026; Wang S. et al., 2024). This is due to their excellent biocompatibility, injectability, and robust drug delivery capabilities. Antibiotics and growth factors (GFs) can be co-loaded into the hydrogel matrix. Their release behaviors can be regulated through structural design and cross-linking methods. This strategy is expected to establish a more logical therapeutic rhythm between early antibacterial action and subsequent osteopromotion. Consequently, it provides new insights for the integrated repair of IBDs (Dini et al., 2026).

Bifunctional hydrogels have increasingly become a primary focus in the research of repair materials for IBDs. This trend centers on the sequential synergistic goal of achieving “early infection control followed by bone regeneration.” Previous studies have achieved the sequential release of vancomycin and bone morphogenetic protein-2 (BMP-2) through various methods. These methods include microspheres, hydrogel networks, and peptide grafting. The feasibility of simultaneously advancing infection control and bone repair has been successfully verified *in vitro* and in animal models (Cui et al., 2026; Zhan et al., 2024). Meanwhile, researchers are actively integrating functional modules, such as nanozymes and magnetic components, into hydrogel systems. This integration aims to further enhance their capabilities for hemostasis, imaging, antibacterial action, and regeneration regulation (Chen et al., 2025b; Shi et al., 2025). These advancements indicate that the field has gradually shifted from simple drug loading to advanced composite designs. These novel designs simultaneously prioritize drug delivery efficiency and cellular microenvironment regulation.

The repair of IBDs does not rely solely on the sequential release of drugs. It is also closely related to microenvironment abnormalities. These abnormalities include local inflammation, oxidative stress, immune cell phenotype transition, and insufficient vascularization. Therefore, the research focus on bifunctional hydrogels is expanding. It has shifted from passive drug delivery to the active regulation of the lesion microenvironment. For instance, researchers have incorporated copper- and strontium-doped mesoporous bioactive glass or photothermal/antioxidant nanocomponents into these systems. These additions can yield multiple benefits. They simultaneously improve antibacterial, immunomodulatory, and osteogenic conditions (Wang S. et al., 2024; Zhan et al., 2026). Although hydrogels have been extensively investigated for infection control and bone regeneration, how antibiotic- and GF-loaded systems achieve staged delivery and coordinated repair in IBDs has not been comprehensively reviewed. Unlike previous reviews that have mainly discussed antibacterial hydrogels, osteogenic biomaterials, or hydrogel-based drug delivery separately, this review specifically focuses on the coordination between antibiotic-mediated infection control and GF-mediated tissue regeneration. Particular attention is given to how spatiotemporal delivery strategies connect infection control with immune microenvironment remodeling, angiogenesis, and osteogenic differentiation. On this basis, this review integrates material design, spatiotemporal release, molecular and cellular mechanisms, preclinical evaluation, and translational challenges within a unified framework for IBD repair.

Relevant literature was identified through searches of PubMed and Web of Science, with emphasis on studies published between 2020 and September 2026, using combinations of terms including “infected bone defect,” “osteomyelitis,” “hydrogel,” “antibiotic,” “growth factor,” “sequential release,” “controlled release,” “bone regeneration,” and “immunomodulation.” Figure 1 summarizes the core elements of this review. It outlines the pathological challenges of IBDs, the design principles of hydrogel carriers, synergistic repair mechanisms, and commonly used evaluation systems.

**FIGURE 1:**
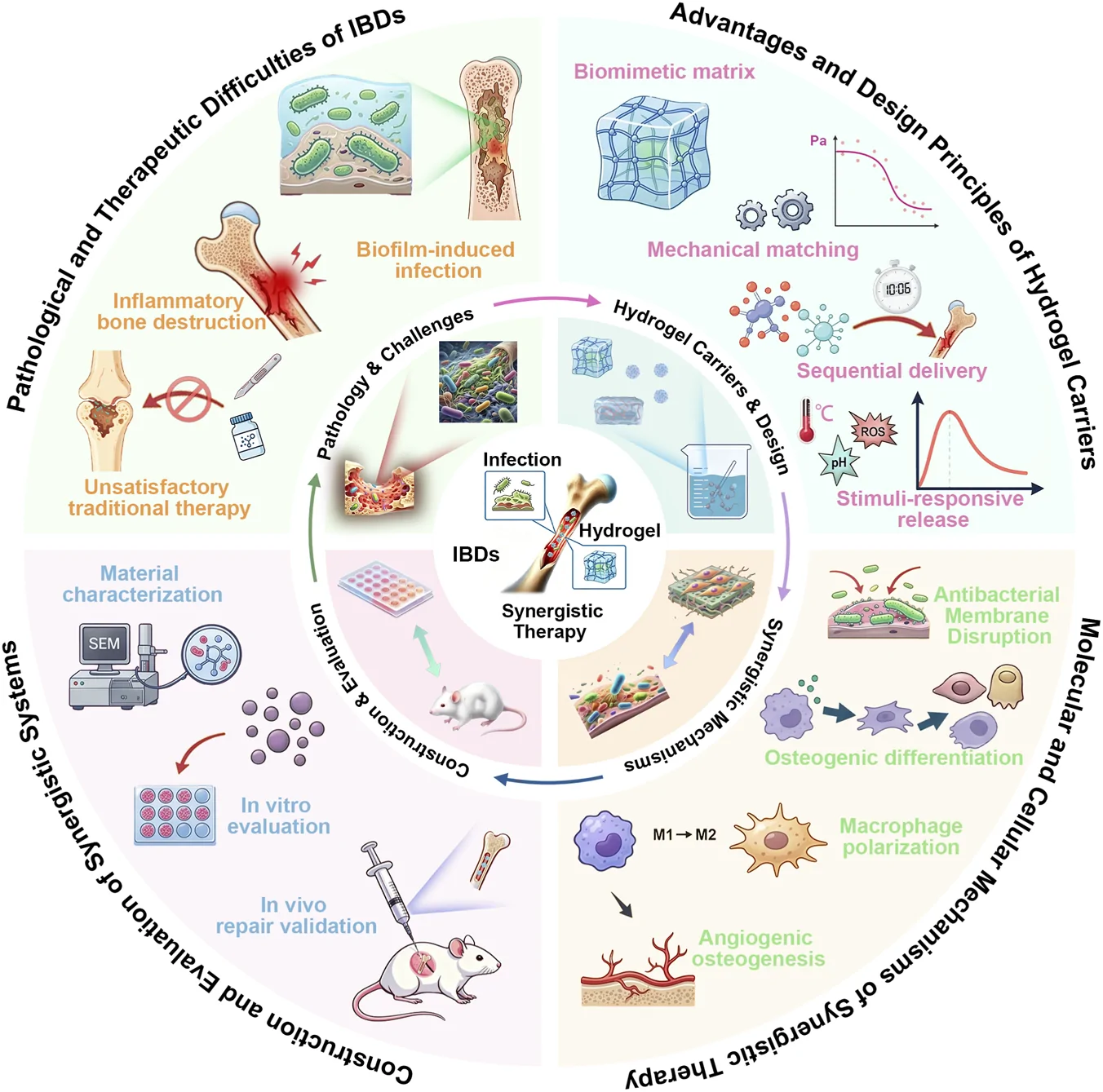
Pathological challenges, design principles, synergistic mechanisms, and evaluation systems of bifunctional hydrogels in the repair of IBDs. This figure summarizes the main pathological challenges of IBDs. These challenges include bacterial colonization, biofilm formation, inflammatory responses, bone destruction, and the limitations of traditional treatments. Bifunctional hydrogels can achieve the local delivery of antibiotics and GFs. This is accomplished through advanced designs, such as biomimetic matrices, mechanical adaptation, sequential release, and stimuli-responsive release. Consequently, these hydrogels promote simultaneous infection control and bone regeneration through antibacterial effects, immunomodulation, angiogenesis, and osteogenic differentiation.

## 2 Pathological characteristics and treatment challenges of IBDs

### 2.1 The vicious cycle of infection and bone defects

The formation of bacterial biofilms is the greatest obstacle in the treatment of IBDs. After colonizing the defect area, bacteria initially secrete various extracellular matrix (ECM) components, such as polysaccharides. Subsequently, they gradually form structurally complex biofilms (Ying et al., 2025). Biofilm formation not only attenuates the host’s immune clearance of bacteria. It also hinders the penetration of antibiotics into the lesion, which reduces their bactericidal efficacy. Ultimately, this progression causes the infection to develop into a chronic and refractory state (Cao et al., 2024). Persistent bacterial infection can induce the massive release of pro-inflammatory cytokines. These include tumor necrosis factor-alpha (TNF-α), interleukin-1 beta (IL-1β), and interleukin-6 (IL-6). Consequently, this triggers a severe inflammatory response at the bone defect site. On the one hand, these inflammatory signals inhibit osteoblast function, leading to impaired bone formation. On the other hand, they activate osteoclasts, resulting in increased bone resorption. Ultimately, infection and bone defects mutually reinforce each other. This forms a continuously worsening vicious cycle (Campbell et al., 2024). A study by Chen Y et al. (2025) pointed out a critical mechanism in osteomyelitis caused by *S. aureus* (*Staphylococcus aureus*). They found that increased osteoclast activation mediated by RANKL is a major cause of bone loss. Furthermore, Yang Q et al. (2025) indicated that the infection-induced inflammatory response can cause local microvascular damage and microcirculation disorders. This disrupts the supply of oxygen and nutrients. It also restricts endogenous repair cells, such as mesenchymal stem cells (MSCs), and systematically administered drugs from reaching the lesion site. Consequently, bone regeneration is further impeded. Therefore, systematic and synergistic interventions are necessary to effectively control infection and repair bone defects. These interventions must simultaneously target three key aspects: infection control, inflammation regulation, and the reconstruction of bone repair capacity.

### 2.2 Limitations of traditional staged treatments

Currently, the clinical treatment for IBDs remains primarily staged. The first stage involves thorough surgical debridement and the resection of necrotic bone tissue. The defect is then filled with antibiotic-loaded bone cement. Later, once the infection is stably controlled, a second stage is performed. This stage reconstructs the bone defect site through bone grafting or bone transport techniques. However, this therapeutic strategy still faces numerous limitations. First, the increased number of surgeries in staged treatments prolongs hospitalization and increases financial burdens. Furthermore, it imposes significant psychological and physiological stress on patients (Shen et al., 2023). The antibiotic bone cement implanted during the first stage can exert local anti-infection effects. However, its drug release profile is characterized by an initial burst release followed by a rapid decay. This makes it difficult to maintain a stable, controllable, and long-term antibacterial concentration. Excessively high local drug concentrations in the early stage may increase the risk of cytotoxicity. Subsequently, the rapid decay leads to a sudden drop in anti-infection capacity. This hinders the effective clearance of bacterial biofilms. Moreover, this abrupt drug release pattern easily promotes the development of bacterial resistance (Vandenbulcke et al., 2025). Additionally, bone cement itself lacks osteopromotive functions. Therefore, after the removal of the antibiotic bone cement in the second stage, the treatment of the residual defect still relies entirely on additional bone reconstruction methods (Qiao et al., 2022). Moreover, the optimal surgical timing for second-stage bone grafting is difficult to determine precisely. If bone grafting is performed before the infection is fully eradicated, it easily leads to infection recurrence. Conversely, a prolonged delay in bone grafting after infection control may cause local soft tissue contracture and atrophy. This significantly increases the risk of nonunion (Shen et al., 2024). Studies have indicated that extensive bone defects and repeated debridement during induced membrane technique treatments are closely associated with an elevated risk of nonunion and infection recurrence. Both can be considered important independent risk factors (Shen et al., 2024). All these factors increase the uncertainty of outcomes in traditional staged treatments. Therefore, it is necessary to develop integrated treatment strategies that can simultaneously address both infection control and bone regeneration.

## 3 Advantages and design principles of functionalized hydrogels as synergistic treatment carriers

### 3.1 Adaptation of physicochemical properties and biological functions of hydrogels

Hydrogels are a class of biomaterials characterized by high water content and a three-dimensional spatial network structure. Their internal environment closely resembles the ECM. Therefore, they can provide an excellent local microenvironment for cell adhesion, migration, and proliferation (Bai L. et al., 2023). The inherent swelling behavior and adjustable mechanical properties of hydrogels can be regulated. This is achieved by modifying the cross-linking density, polymer composition, and composite reinforcement strategies. Consequently, these modifications allow the hydrogels to match the structural support conditions required for bone repair within a certain range (Bai L. et al., 2023). Previous studies have shown that the porous and highly hydrated nature of hydrogels facilitates the diffusion of oxygen and nutrients. Furthermore, optimizing the base materials and cross-linking methods can further enhance their mechanical adaptability (Chen S. et al., 2025a; Yu et al., 2024; Yu et al., 2022). Therefore, these adjustable mechanical properties and structural features—which are highly similar to natural tissues—are essential prerequisites. They make hydrogels an ideal adaptable material for bone tissue applications.

Hydrogels are commonly used as drug delivery carriers because they possess a well-developed internal porous network structure. They can load antibiotics and GFs, such as vancomycin, rifampin, BMP-2, and vascular endothelial growth factor (VEGF). This is achieved through physical encapsulation, chemical conjugation, or electrostatic interactions (Li X. et al., 2024). For IBDs, this drug-loading capacity is of great significance. It provides a theoretical basis for the simultaneous execution of local anti-infection and osteopromotion. A study by Zuo et al. (2022) demonstrated this potential. They found that supramolecular hydrogels constructed from amphiphilic peptides could co-assemble with osteogenic growth peptide (OGP). This achieved a relatively stable and sustained release of OGP. Consequently, it exhibited excellent efficacy in promoting the regeneration of cranial bone defects. Wang et al. (2025b) reported on a photocrosslinked GelMA/HAMA composite hydrogel loaded with BMP-2 and black phosphorus nanosheets. This hydrogel could maintain the stable release of BMP-2. Furthermore, it promoted the recruitment and osteogenic differentiation of MSCs. Another significant advantage of hydrogels as carriers lies in the adjustability of their release behaviors. Lavanya et al. (2020) designed a temperature/pH-responsive chitosan-based injectable hydrogel. This hydrogel features high water content, minimally invasive administration, a porous network structure, and excellent adaptability to irregular defects. Therefore, it has shown promising application prospects in bone tissue engineering. Furthermore, the degradation rate of hydrogels can be regulated by selecting different types of base materials. These materials include gelatin methacryloyl (GelMA), polyethylene glycol (PEG), polyamino acids, or sodium alginate. This selection can be combined with various construction methods, such as photocrosslinking, enzymatic crosslinking, or ionic crosslinking. Consequently, the degradation rate can better match the drug release process and the natural rhythm of bone repair (Zhu et al., 2024). For example, Sreekumaran et al. (2021) designed a photocrosslinked GelMA/polyethylene glycol diacrylate hydrogel incorporated with nano-hydroxyapatite. This material exhibited excellent properties in terms of gel formation, swelling, degradation, and internal structural integrity. Therefore, it is considered highly suitable for bone tissue engineering applications. By rationally designing the material degradation and drug release processes, a sequential treatment concept can be realized. This approach achieves the rapid early release of antibiotics to efficiently control infection. Subsequently, it allows for the sustained and slow release of GFs to promote osteogenic repair.

Importantly, these physicochemical properties are not independent material parameters but directly influence therapeutic outcomes. Cross-linking density and network mesh size regulate molecular diffusion and therefore affect the release kinetics of antibiotics and GFs. Excessive cross-linking may improve structural stability and prolong drug retention but restrict molecular diffusion and cell infiltration, whereas insufficient cross-linking may result in burst release and premature structural collapse. Similarly, hydrogel degradation should be coordinated with tissue regeneration: rapid degradation may compromise local drug retention and mechanical support, while excessively slow degradation may hinder tissue ingrowth and remodeling. Mechanical stiffness and porosity also affect cell adhesion, migration, nutrient transport, and osteogenic differentiation. Thus, therapeutic performance depends on balancing drug release, degradation, mechanical stability, and biological accessibility rather than maximizing any single material property.

### 3.2 Design of sequential and synergistic release strategies

The sequential release of antibiotics and GFs is the core of bifunctional hydrogel design. The overarching principle is to rapidly establish an effective antibacterial concentration during the early stage of infection. Subsequently, once the infection is controlled, a sustained output of osteogenic-related signals must be maintained (Cui et al., 2026). Compared with simultaneous release, sequential delivery may better match the changing biological requirements of IBD repair. During the early phase, rapid antibiotic release is required to reduce the bacterial burden and disrupt biofilms, whereas premature GF delivery may provide limited regenerative benefit in a highly infected and inflammatory microenvironment. After infection and inflammation are alleviated, sustained GF delivery can more effectively support cell recruitment, angiogenesis, and osteogenic differentiation. Thus, temporal separation of antibacterial and regenerative signals may improve the coordination between infection control and subsequent tissue regeneration. To achieve this goal, researchers have adopted several strategies. These include encapsulation within hydrogel microspheres (HMs), network stratification, and covalent grafting. For instance, Cui et al. constructed an engineered poly(amino acid) hydrogel. By loading vancomycin and grafting BMP-2 peptides, they successfully achieved synergy between early antibacterial action and later-stage osteopromotion (Cui et al., 2026). Similarly, antibiotics can be encapsulated within easily degradable HMs in the early stage. Meanwhile, GFs can be immobilized within the primary hydrogel network. This structural design can generate a much clearer release gradient within a single system.

The concept of spatially distributed synergistic design partially originates from the development of manufacturing technologies such as 3D printing. This design approach involves constructing scaffolds with porous gradient characteristics. It separately loads antibiotics and GFs into different regions. Consequently, it achieves distinct functions within the material. Zhang Q et al. (2023) successfully constructed a bifunctional composite scaffold. They achieved this by combining a fingolimod-loaded 3D-printed polycaprolactone (PCL) scaffold with a vancomycin-loaded aldehyde hyaluronic acid/carboxymethyl chitosan hydrogel. The 3D-printed scaffold primarily provides mechanical support, which contributes to angiogenesis and bone tissue regeneration. Meanwhile, the hydrogel filled within the pores exerts a local antibacterial effect. Further spatially distributed synergistic design can load antibiotics on the outer layer of the scaffold. This allows the antibiotics to directly contact the infected tissue. Simultaneously, GFs are concentrated in the inner core region designed for bone regeneration. Such a design forms an antibacterial defense line containing antibiotics at the periphery of the material. This effectively restricts the infection from spreading inward. At the same time, it forms a relatively stable local environment rich in growth regulatory signals inside the material. This internal environment exerts an osteopromotive effect after the infection is stably controlled. Recently, a study successfully fabricated a biocomposite scaffold with a biomimetic woven pattern. They utilized dual-nozzle synergistic 3D bioprinting technology (Yin et al., 2026). Its structure effectively combines soft hydrogel filaments with rigid PCL-based filaments. The hydrogel portion is loaded with factors related to angiogenesis and osteogenesis. Meanwhile, the PCL matrix is incorporated with magnesium oxide (MgO) nanoparticles. This PCL matrix not only possesses superior mechanical properties but also continuously and controllably releases magnesium ions (Mg^2+^). This design may facilitate the spatial coordination of angiogenesis and bone formation. This multi-material synergistic design provides new possibilities for the integration of infection control and bone regeneration.

Stimuli-responsive release strategies emphasize “on-demand drug delivery.” Infected lesions are often accompanied by specific characteristics. These include decreased pH, elevated bacteria-related enzymes, and the abnormal accumulation of reactive oxygen species (ROS). Therefore, these signals can serve as triggering conditions to design smart hydrogels (Zhu et al., 2024). For example, conductive or ROS-responsive hydrogels can regulate drug release in response to local environmental changes. Furthermore, they may simultaneously participate in antibacterial action and immune microenvironment regulation (Yang Q. et al., 2025; Zhang et al., 2024). Compared with constant drug release, these strategies are more effective in improving local drug delivery efficiency. They also significantly reduce drug exposure in non-lesion areas.

Overall, the key to bifunctional hydrogels does not lie in the simple co-loading of two types of drugs. Rather, it relies on three major design principles: temporal, spatial, and stimuli-responsive designs. These advanced designs ensure that antibiotics and GFs exert their effects at the appropriate stages and in specific locations. Common construction modes include physical encapsulation, chemical conjugation, combination with micro/nanocarriers, and 3D-printed scaffold structures. Additionally, they involve release mechanisms that are responsive to pH, ROS, enzymes, and temperature. Figure 2 summarizes the aforementioned strategies. Building on the design principles outlined above, Table 1 summarizes the major design strategies reported for dual-functional hydrogels in infected bone defect repair, highlighting representative hydrogel systems, therapeutic payloads, key advantages, and remaining limitations.

**FIGURE 2:**
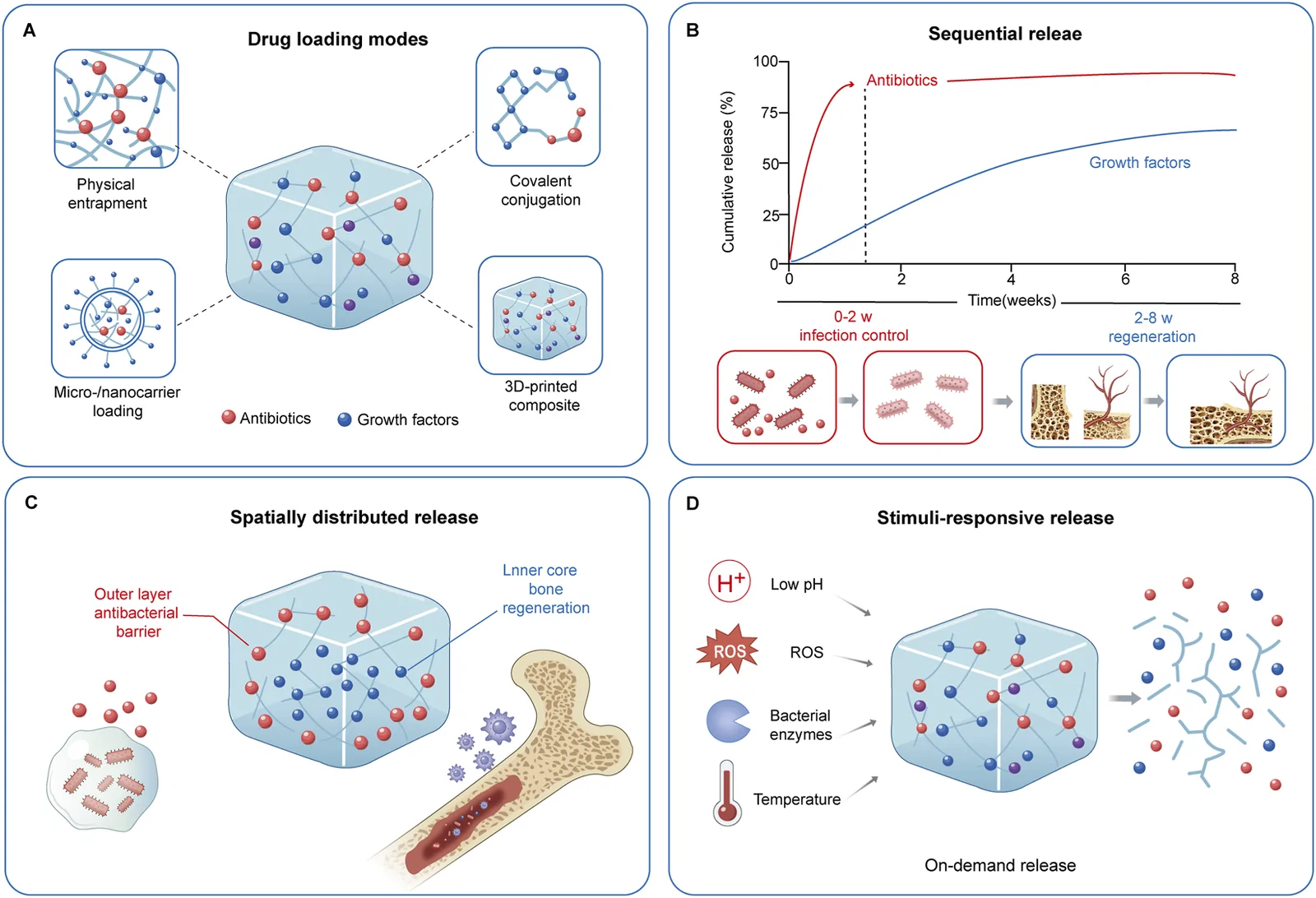
Construction modes and spatiotemporally controllable release strategies of bifunctional hydrogels. **(A)** Major drug-loading modes, including physical encapsulation, micro/nanocarrier loading, covalent conjugation, and 3D-printed composite structures. **(B)** Sequential release strategy, with early antibiotic release for infection control followed by sustained growth factor release to promote bone regeneration. **(C)** Spatially distributed release strategy, with antibiotics located in the outer region to provide an antibacterial barrier and growth factors concentrated in the inner region to support bone regeneration. **(D)** Stimuli-responsive release strategy triggered by pathological signals such as low pH, reactive oxygen species (ROS), bacterial enzymes, or temperature.

**TABLE 1:** Main design strategies of bifunctional hydrogels for the repair of IBDs.

Design strategies	Representative hydrogels	Main loaded components	Advantages	Disadvantages	References
Sequential release hydrogels	Poly(amino acid), chitosan/nano-hydroxyapatite hydrogels	Antibiotics, BMP-2, or BMP-2 mimetic peptides	Early antibacterial action, later osteopromotion	Release profiles still require precise optimization	(Cui et al., 2026; Zhan et al., 2024)
Micro/nanocarrier composite hydrogels	Hydrogels composited with PLGA microspheres, nanoparticles, or liposomes	Antibiotics, GFs, or small-molecule drugs	Improve drug stability and prolong release	Complex manufacturing, long-term biosafety remains to be verified	(Lee et al., 2022; Tao et al., 2020)
Scaffold-hydrogel composite systems	3D-printed scaffolds combined with drug-loaded hydrogels	Antibiotics, BMP-2, FTY720, etc.	Provide both mechanical support and local drug delivery	Complex systems, relatively high difficulty in translation	(Lee et al., 2022; Zhang Q. et al., 2023)
Stimuli-responsive hydrogels	pH-, ROS-, enzyme-, or temperature-responsive hydrogels	Antibacterial drugs, antioxidant components, GFs	On-demand drug release according to the infected microenvironment	Insufficient *in vivo* response thresholds and precision	(Lavanya et al., 2020; Zhang et al., 2024)
Immunomodulatory hydrogels	Hydrogels composited with ions, nanozymes, or conductive materials	*Cu* ^*2+*^, *Zn* ^*2+*^, *Ca* ^*2+*^, or immunomodulatory components	Regulate inflammatory responses and M1/M2 polarization	Long-term immunological effects remain unclear	(Jin et al., 2026; Xu et al., 2023)
Cell/exosome-combined hydrogels	Hydrogels composited with BMSC sheets, exosomes, or bioactive molecules	Antibiotics, cytokines, or exosomes	Enhance osteogenesis, vascularization, and immune repair	High difficulty in standardized manufacturing and regulation	(Wang et al., 2022; Zhao et al., 2023)

These hydrogel systems differ in their capacities for release control, mechanical support, microenvironmental responsiveness, and immunomodulation. Micro/nanocarrier-based systems are particularly suitable for improving drug stability and controlling release kinetics, whereas scaffold–hydrogel composites provide greater structural and mechanical support for larger bone defects. Stimuli-responsive hydrogels offer advantages in adapting drug release to changes in the infected microenvironment, while immunomodulatory systems are designed to actively regulate inflammatory and osteoimmune responses. Therefore, the optimal strategy should be selected according to the dominant therapeutic requirement and defect characteristics rather than relying on a single design for all clinical scenarios. Importantly, increasing structural complexity does not necessarily translate into superior therapeutic performance. Although multicomponent systems can provide more precise release control and additional biological functions, they may also increase variability in drug loading, degradation behavior, batch-to-batch reproducibility, and manufacturing. Conversely, simpler hydrogel systems may offer greater reproducibility and translational feasibility but provide less precise control over spatial or temporal drug delivery. Hydrogel design should therefore balance functional sophistication with structural simplicity, reproducibility, and clinical feasibility. Ultimately, their therapeutic relevance in IBD repair depends on whether these features can be integrated to coordinate infection control, immune microenvironment remodeling, angiogenesis, and osteogenic differentiation. Accordingly, the next section discusses the reparative effects of antibiotic- and GF-loaded hydrogels from molecular and cellular perspectives.

## 4 Molecular and cellular mechanisms of the synergistic effects of antibiotics and GFs

The therapeutic synergy between antibiotics and GFs extends beyond the simple addition of antibacterial and osteogenic effects. Persistent bacterial burden, biofilms, inflammatory cytokines, and excessive ROS can impair MSC survival, suppress osteogenic differentiation, and compromise vascular regeneration. Early antibiotic delivery therefore not only reduces the bacterial burden and disrupts biofilms but also removes major biological constraints on subsequent tissue regeneration. As infection and excessive inflammation subside, sustained GF signaling can more effectively promote cell recruitment, angiogenesis, and osteogenic differentiation within a more permissive microenvironment. In this context, infection control and tissue regeneration are biologically interdependent rather than independent therapeutic processes. The synergistic effect of bifunctional hydrogels therefore arises from the temporal coupling of pathogen clearance, microenvironment normalization, and regenerative signaling. Based on this progression, the mechanism can be summarized into two interconnected phases: early infection control and alleviation of osteogenic inhibition, followed by immunomodulation, angiogenesis, and osteogenic regeneration. Figure 3 illustrates this overall process.

**FIGURE 3:**
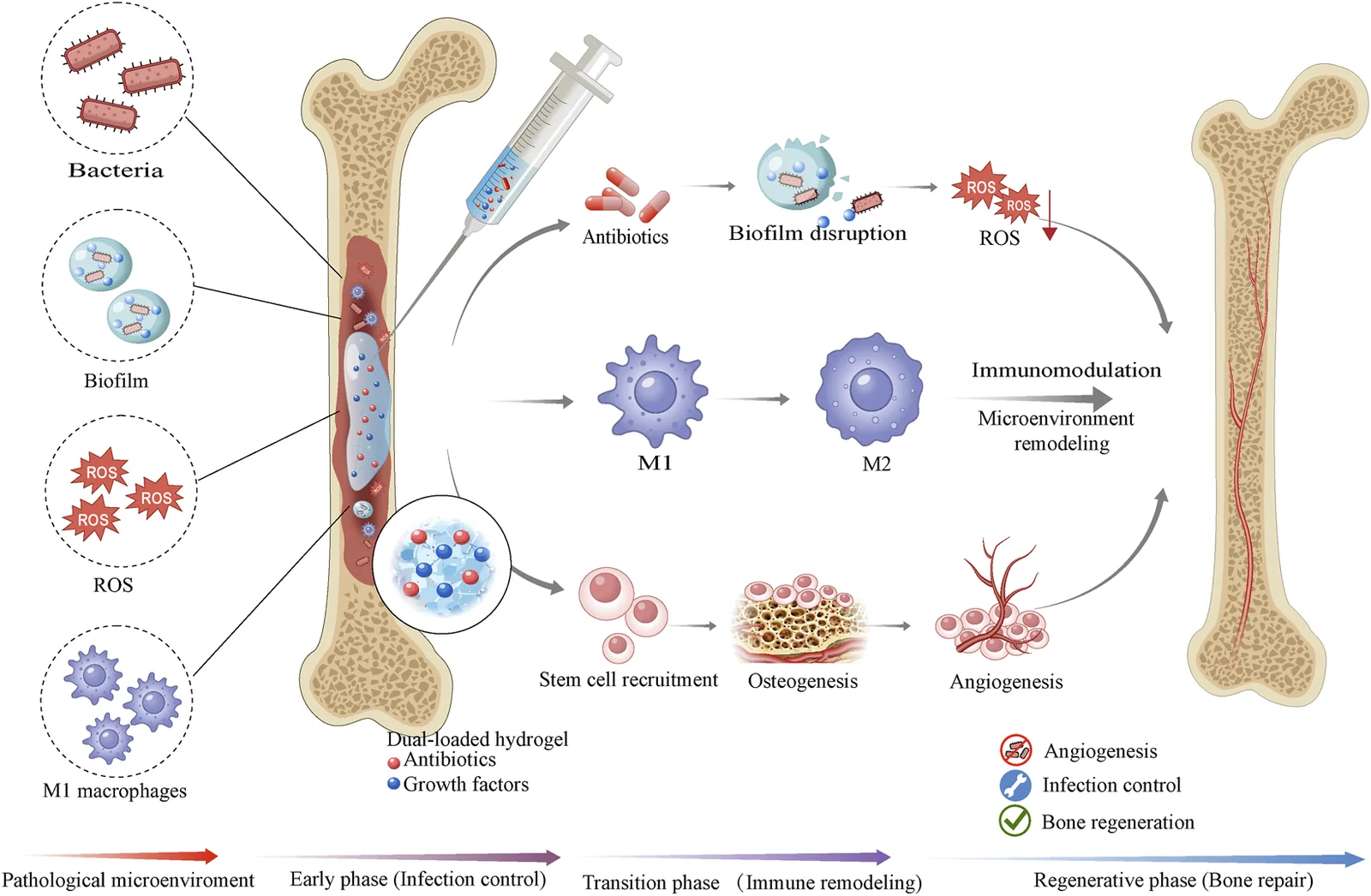
Synergistic mechanisms of antibiotic and GF-loaded hydrogels for the repair of IBDs. In the early stage, bifunctional hydrogels release antibacterial components. This process reduces the bacterial burden, disrupts biofilms, and alleviates inflammation and oxidative stress. Subsequently, these hydrogels promote the transition of the local immune microenvironment. They shift it from a pro-inflammatory state to a pro-repair state. This transition induces the polarization of M1 macrophages toward the M2 phenotype. In the later stage, the continuous release of GFs promotes stem cell recruitment, osteogenic differentiation, angiogenesis, and matrix mineralization. Ultimately, this achieves the synergistic integration of infection control and bone regeneration.

### 4.1 Infection control and alleviation of osteogenic inhibition

In the repair of IBDs, it is first necessary to break the adverse microenvironment. This environment is mutually maintained by bacteria, biofilms, and persistent inflammation. Bacterial biofilms attenuate host immune clearance and hinder antibiotic penetration. Consequently, this causes the infection to become prolonged and refractory (Lin et al., 2025). Therefore, the focus of early treatment is to rapidly reduce the pathogen burden and weaken the biofilm barrier. Relevant studies indicate that sonodynamic or pH/ROS-responsive strategies can enhance local antibacterial effects. Furthermore, they can alleviate persistent inflammation to a certain extent (Yan et al., 2026). As the bacterial load decreases, the levels of pro-inflammatory cytokines such as TNF-α and IL-1β also decline. Subsequently, the inhibition exerted on osteoblasts and MSCs is significantly alleviated (Yan et al., 2026). Simultaneously, neutrophils and M1 macrophages reduce their continuous release of ROS and proteolytic enzymes. This reduction helps protect the newly formed bone matrix and the drug delivery system itself (Sun J. et al., 2024). Therefore, early infection control is not merely a bacteriostatic process. It is an essential prerequisite for the initiation of subsequent bone regeneration.

### 4.2 Synergy of immunomodulation, angiogenesis, and osteogenic differentiation

After the infection is effectively controlled, GF-mediated regenerative signals and immune microenvironment remodeling become the dominant factors in the repair process. Osteogenic factors, represented by BMP-2, can activate Smad-related pathways. This process promotes the expression of key transcription factors such as Runx2 and Osterix. Consequently, it enhances alkaline phosphatase activity, matrix secretion, and mineralized nodule formation (Lou et al., 2025; Wang et al., 2026). Different GFs may serve complementary roles during this regenerative phase. BMP-2 primarily promotes osteogenic commitment and differentiation of progenitor cells through BMP/Smad signaling, whereas VEGF mainly supports endothelial cell recruitment, proliferation, and neovascularization. Because newly formed vessels provide oxygen, nutrients, and cellular support for regenerating bone, angiogenic and osteogenic signals are closely coupled. Therefore, the selection or combination of GFs should consider the specific biological requirements at different stages of IBD repair. Furthermore, some antibiotics at sub-inhibitory concentrations may exert an auxiliary effect on the osteogenic process. This is achieved by inhibiting matrix metalloproteinases (MMPs) or alleviating inflammatory responses (Li Q. et al., 2024). More importantly, after the infection is alleviated, the local immune state typically shifts from a pro-inflammatory phase to a pro-repair phase. Macrophages gradually polarize from the M1 to the M2 phenotype (Jin et al., 2026). These M2 macrophages subsequently release various factors, including interleukin-10 (IL-10), VEGF, and platelet-derived growth factor (PDGF). It should be noted that the M1/M2 classification represents a simplified framework for describing macrophage polarization. *In vivo*, macrophages exhibit substantial phenotypic plasticity and may adopt intermediate or mixed activation states in response to dynamic signals within the infected and regenerating microenvironment. Therefore, the M1-to-M2 transition discussed here should be interpreted as a general shift from a predominantly pro-inflammatory toward a more pro-reparative phenotype rather than as a strictly binary conversion. Immune regulation in IBD repair also extends beyond macrophage polarization alone. Crosstalk among macrophages, MSCs, endothelial cells, and biomaterial-derived signals can influence both infection control and subsequent tissue regeneration. Macrophage-derived cytokines and growth signals can regulate MSC recruitment and osteogenic differentiation as well as endothelial activation, whereas changes in ROS levels, ion concentrations, and matrix properties generated by functional hydrogels can in turn modify immune-cell behavior. Recent biomaterial strategies therefore increasingly aim to coordinate multiple cellular compartments rather than targeting a single macrophage phenotype. For example, an infection-responsive biomaterial interface was recently shown to coordinate macrophage reprogramming with BMSC recruitment and Wnt-associated osteogenic signaling, enabling endogenous immune and stem cells to contribute jointly to bacterial clearance and bone formation (Li Z. et al., 2026). Such multicellular regulation may provide a more physiologically relevant basis for coupling inflammation resolution with angiogenesis and osteogenesis. This secretion creates favorable conditions for inflammation resolution, angiogenesis, and matrix reconstruction (He et al., 2026). Therefore, the later-stage effects of bifunctional hydrogels are not limited to merely promoting bone formation. Instead, they jointly enhance repair efficiency through the active coupling of immunomodulation, angiogenesis, and osteogenic differentiation (Chen et al., 2025a; Liu et al., 2025; Su et al., 2025; Xu et al., 2023). The simultaneous loading of antibiotics and GFs via hydrogels not only alleviates the inhibitory effect of infection on bone formation. It also utilizes immunomodulation and GF signaling to cooperatively improve the local repair environment. This provides more favorable conditions for angiogenesis and osteogenic differentiation. Ultimately, it significantly enhances the efficiency and quality of bone regeneration.

## 5 Construction and evaluation of hydrogel-based synergistic treatment systems

Whether bifunctional hydrogels can truly be applied in the repair of IBDs cannot be judged solely based on material design concepts. A systematic chain of evidence must be established across three levels. These levels include material characterization, *in vitro* functional verification, and *in vivo* animal experiments. Figure 4 summarizes the construction and evaluation workflow of these systems.

**FIGURE 4:**
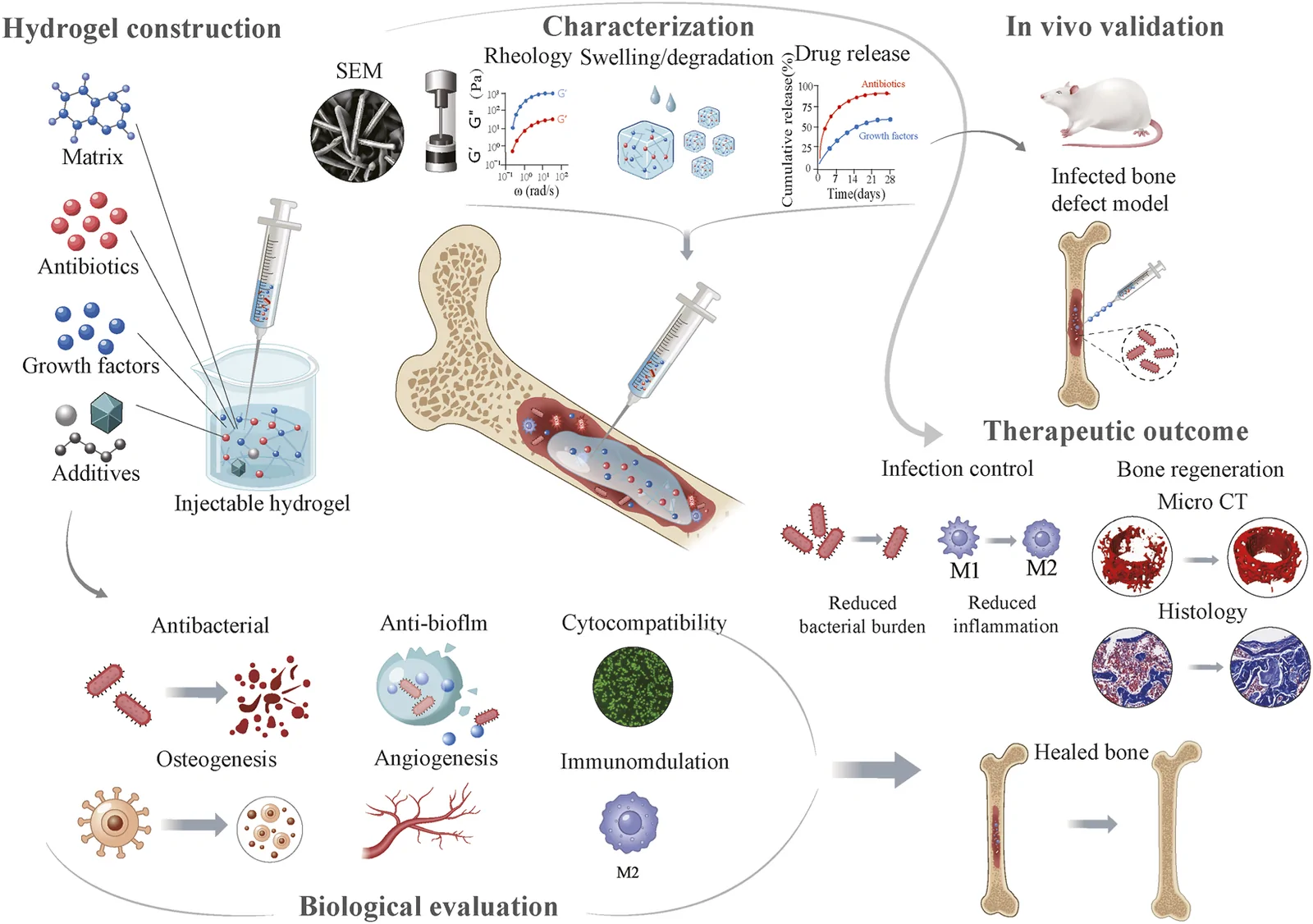
Construction and evaluation workflow of hydrogels for the repair of IBDs. This figure summarizes the basic workflow of bifunctional hydrogels, ranging from material construction to *in vitro* and *in vivo* validation. The hydrogel system typically consists of a polymer matrix, antibiotics, GFs, and specific functional components. The material is thoroughly characterized through its morphological structure, rheological properties, swelling/degradation behaviors, and drug release profiles. Furthermore, its functional evaluation includes antibacterial activity, anti-biofilm properties, cytocompatibility, osteogenesis, angiogenesis, immunomodulation, and overall repair efficacy in animal models.

### 5.1 Examples of material system construction and performance characterization

Polymer-based hydrogels have received extensive attention in the construction of sequential treatment platforms. This is due to their highly designable molecular structures and excellent biocompatibility. For instance, poly(ethylene glycol)-poly(L-alanine-co-L-phenylalanine) hydrogels, synthesized via the ring-opening polymerization of N-carboxyanhydrides, exhibit excellent injectability (Guo et al., 2024). Based on this polymer, a bifunctional hydrogel can be constructed by grafting BMP-2 mimetic peptides and loading vancomycin (Zhang Z. et al., 2023). This hydrogel possesses both antibacterial and osteogenic functions, featuring sequential release characteristics. The rationale behind this design is to maintain local shape-forming ability and structural stability. Simultaneously, it achieves the synergistic loading and staged release of antibiotics and GFs (Cui et al., 2022). Compared with single-function hydrogel systems, composite hydrogels are more suitable for constructing complex spatiotemporal drug delivery platforms. This is because they can simultaneously leverage the advantages of different components. A representative study was conducted by Lee et al. (2022). They combined antibiotic-loaded (such as rifampin or moxifloxacin) poly(lactic-co-glycolic acid) (PLGA) microspheres with BMP-2-loaded GelMA hydrogels. This combination was used to modify the surface of 3D-printed titanium alloy scaffolds. In this composite system, the GelMA hydrogel functions as a drug carrier and a matrix for cell adhesion and growth. Meanwhile, the PLGA microspheres precisely regulate antibiotic release through their degradable properties. Therefore, the material itself serves as a drug delivery platform that effectively integrates the two-stage requirements of early antibacterial action and later osteopromotion (Zhao et al., 2025). Furthermore, composite systems based on natural polymers have also shown promising application prospects. Wang et al. (2022) constructed a combined system. They integrated a vancomycin-loaded calcium sulfate hemihydrate/nano-hydroxyapatite/carboxymethyl chitosan hydrogel with MSC sheets. This system can achieve local antibiotic delivery. It also provides a supportive structure conducive to osteogenesis for the cells.

Evaluating the clinical application prospects of functionalized hydrogels requires systematic performance characterization. In particular, basic physicochemical indicators must be carefully examined. These include the swelling ratio, degradation behavior, and mechanical strength. These factors directly affect the stable presence and supporting capacity of the material in the defect area (Dong et al., 2025). Additionally, parameters such as the encapsulation efficiency, drug loading capacity, and release profiles of antibiotics (e.g., vancomycin) and GFs (e.g., BMP-2) should be emphatically investigated. This is essential for studying pharmacokinetics (Tao et al., 2020). An ideal release pattern typically involves releasing a sufficient amount of the drug in the early stage. This rapidly brings the local antibiotic concentration to an effective bacteriostatic level. Subsequently, it enters a relatively stable, sustained release phase. This maintains a continuous therapeutic effect and reduces the risk of bacterial resistance (Wu M. Y. et al., 2025). The rationality of the material design can only be fully validated when these pharmacokinetic parameters correspond with subsequent outcomes. These outcomes include antibacterial efficacy, osteogenesis, and *in vivo* repair results. At the *in vitro* level, researchers typically combine antibacterial assays, cytocompatibility evaluations, and osteogenesis-related tests. These comprehensive assessments provide a solid foundation for subsequent animal experiments (Zhou et al., 2024).

### 5.2 *In Vitro* and *in vivo* model validation and efficacy evaluation

Before functionalized hydrogels can enter clinical translation, their repair performance must be systematically verified. This validation relies on comprehensive *in vitro* and *in vivo* models. *In vitro* evaluation is an indispensable step in this process. Common methods include antibacterial assays targeting *S. aureus*, *E. coli* (*Escherichia coli*), or drug-resistant bacterial strains. Additionally, they involve osteogenesis-related tests utilizing MSCs (Chen et al., 2022; Hou et al., 2022; Ren Y. et al., 2025; Suneetha et al., 2023; Yu et al., 2026). During this stage, the ability of hydrogels to promote osteogenic differentiation can be comprehensively assessed. This assessment utilizes alkaline phosphatase (ALP) activity assays and Alizarin Red staining to observe the formation of calcified nodules. Furthermore, it involves analyzing the expression of key osteogenesis-related genes, such as RUNX2, osteopontin (OPN), and osteocalcin (OCN). Some studies have also attempted to establish co-culture systems of bacteria and host cells. These systems simulate the complex environment of IBDs, where pathogens and host cells coexist. Consequently, they provide a more accurate evaluation of the material’s capacity to simultaneously balance anti-infection and osteopromotive functions (Deng et al., 2025).

*In vivo* animal models remain an indispensable basis for evaluating the efficacy and safety of therapeutic strategies (Bai S. et al., 2023). In *in vivo* studies, models of IBDs in the radius, femur, and tibia of rabbits or rats are widely applied. The clinical pathological process of IBDs is simulated by inoculating pathogens, such as *S. aureus*, into the bone defect site and subsequently implanting the material to be evaluated. Postoperative evaluation typically needs to be continuously conducted across multiple levels. Imaging is the most commonly used and mature method among these. X-ray examinations can be used to dynamically observe bone healing trends and implant conditions (Yadav et al., 2023). Furthermore, Micro-CT can achieve 3D reconstruction and quantitative analysis. This allows for a more accurate assessment of new bone formation, bone mineral density, trabecular structural parameters, and the degree of defect repair (Sun S. et al., 2024). Histological and immunohistochemical analyses play a crucial role in elucidating microscopic changes during the repair process. Hematoxylin and eosin (H&E) staining is commonly used to observe inflammatory responses, granulation tissue formation, and new bone formation. Additionally, Masson’s trichrome staining or Van Gieson’s staining can further reflect the deposition and maturation status of collagen fibers (Zheng S. et al., 2023). Immunohistochemical staining can be combined with appropriate biomarkers. For example, CD31 is used to evaluate changes related to the vascular endothelium. Meanwhile, OCN is used to assess the maturation of osteoblasts and bone tissue. This approach provides crucial evidence for local angiogenesis and the bone repair process (Niu et al., 2024; Tan et al., 2024; Teimouri et al., 2026; Wang et al., 2023). When evaluating whether the infection is effectively controlled, it is often necessary to conduct quantitative bacterial analyses on bone and soft tissue samples retrieved postoperatively (Sun Q. et al., 2024; Tucker et al., 2025). An engineered poly(amino acid) hydrogel evaluated in a rabbit radial model of infected bone defects showed effective infection control and enhanced bone regeneration. These findings support the feasibility of a single-stage treatment strategy that combines sequential antibacterial activity with subsequent osteogenic stimulation (Chen S. et al., 2025b; Cui et al., 2026). However, the current evaluation framework remains highly heterogeneous across studies. Antibacterial efficacy is assessed using different bacterial strains, inoculum sizes, biofilm models, and endpoints, whereas osteogenic performance is evaluated using variable cell sources, culture conditions, biomarkers, and observation periods. Drug-release profiles are also frequently measured under simplified *in vitro* conditions that do not reproduce the dynamic pH, enzymatic activity, ROS levels, fluid exchange, and protein-rich environment of infected bone. Moreover, antibacterial, immunomodulatory, angiogenic, and osteogenic outcomes are often evaluated separately, making it difficult to determine whether a material truly achieves coordinated therapeutic effects. These methodological differences limit direct comparison among hydrogel systems. Future evaluation should therefore integrate material properties and release kinetics with standardized biological endpoints under conditions that more closely reproduce the infected bone microenvironment. Despite their value in preclinical evaluation, current animal models do not fully reproduce the complexity of clinical IBDs. Most models use standardized bacterial inoculation, often involving a single bacterial strain, together with relatively uniform bone defects. In contrast, clinical infections may involve polymicrobial communities, mature biofilms, drug-resistant organisms, and considerable variability in host conditions. Differences in immune status, comorbidities, defect size, and infection duration may also influence therapeutic outcomes. Future studies should therefore develop more clinically representative infection models to improve the predictive value of preclinical evaluation.

## 6 Current challenges and future directions

Bifunctional hydrogels loaded with antibiotics and GFs have shown great potential in synergizing infection control and bone regeneration. However, they still face multiple obstacles in the transition from laboratory research to clinical applications. These issues encompass bottlenecks at both the material and biological levels. Examples include drug release mismatches, complex infectious microbiomes, the insufficient stability of GFs, and long-term biosafety concerns. Furthermore, they involve practical real-world problems. These include the limited extrapolability of animal models, the lack of unified functional evaluation standards, and unclear pathways for large-scale manufacturing and clinical translation. Simultaneously, future optimization directions have gradually become clear. These directions focus on smart stimuli-responsive release, multi-modal antibacterial strategies, immunomodulation, vascularization, osteogenesis, and standardized preclinical validation. Accordingly, Figure 5 summarizes the key issues and future development pathways in this field. It categorizes these elements across four levels: current bottlenecks, material optimization, preclinical validation, and clinical translation. Table 2 provides a concise overview of these challenges by linking the underlying problems to possible optimization strategies and key translational considerations.

**FIGURE 5:**
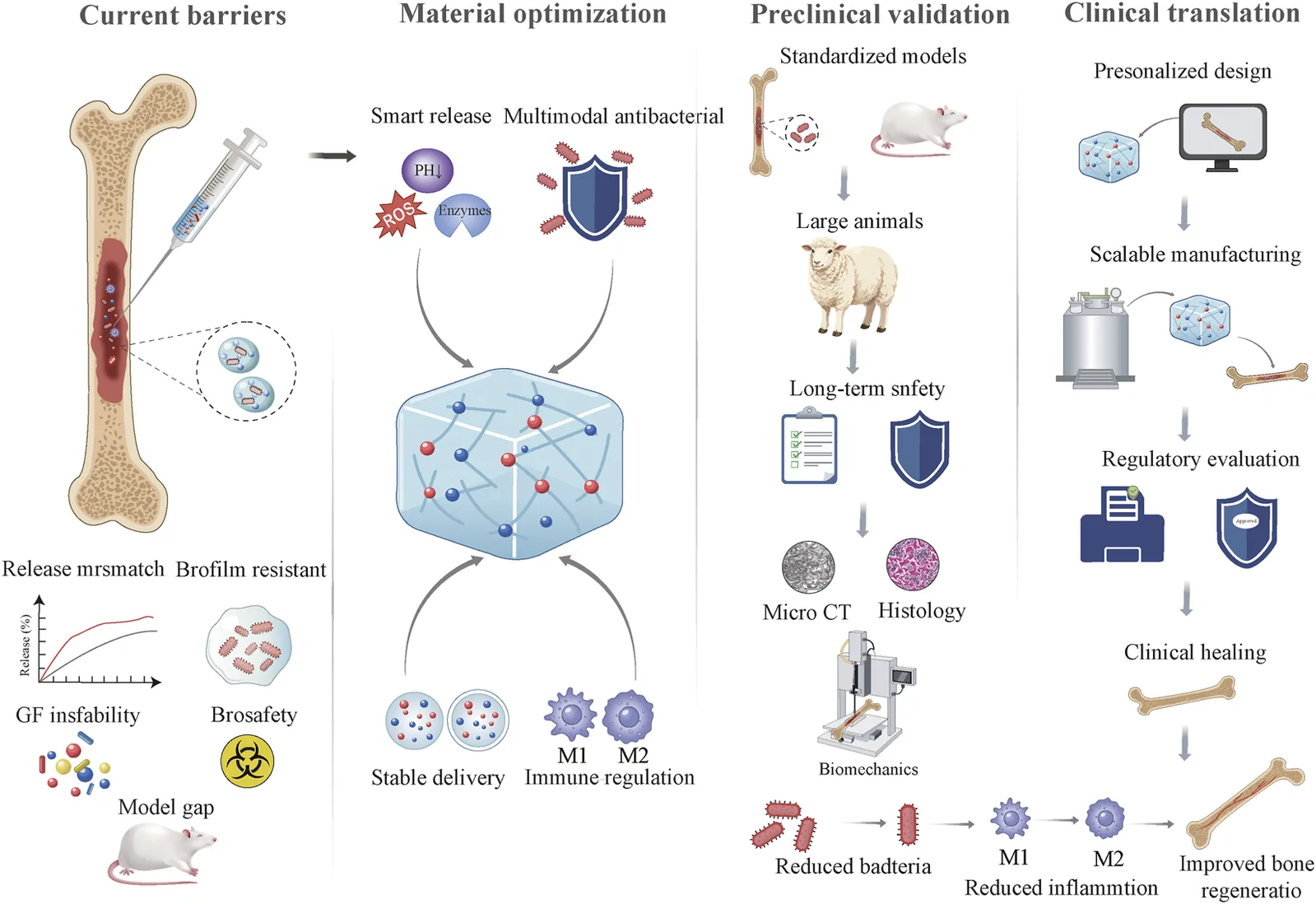
Main challenges, optimization strategies, and translational pathways of bifunctional hydrogels for the treatment of IBDs. This figure summarizes the key issues facing the clinical translation of bifunctional hydrogels. These issues include mismatched release profiles, difficulties in eradicating complex infections, and the insufficient stability of GFs. Additionally, they involve unclear long-term biosafety and the limited extrapolability of animal models. Future optimization directions include smart stimuli-responsive release, multi-modal antibacterial strategies, and stable drug delivery. They also emphasize immunomodulation and the synergy between vascularization and osteogenesis. Furthermore, there is a critical need to enhance standardized models, large animal validation, large-scale manufacturing, and regulatory evaluation.

**TABLE 2:** Main challenges and optimization directions for the clinical translation of bifunctional hydrogels.

Main challenges	Core issues	Optimization directions	Translational focus	References
Mismatched release profiles	Prone to early burst release of antibiotics and inactivation of GFs	Sequential release, micro/nanocarriers, and affinity immobilization	Clarify the optimal release windows for different repair stages	(Wu Y. et al., 2025; Yuan et al., 2022)
Difficulties in eradicating complex infections	Biofilms, drug-resistant bacteria, and polymicrobial infections compromise efficacy	Combinatorial antibacterial strategies, antimicrobial peptides, phages, and photothermal/sonodynamic strategies	Establish infection models more closely resembling clinical scenarios	(Jiang et al., 2025; Xiao et al., 2025)
Inhibited regenerative microenvironment	Inflammation, ROS, and immune imbalances inhibit osteogenesis	Antioxidant, anti-inflammatory, and M1/M2 polarization regulation	Simultaneously evaluate antibacterial, immunomodulatory, and osteogenic effects	(Jin et al., 2026; Wang Z. et al., 2025)
Mismatched mechanical and degradation properties	Insufficient support from pure hydrogels, and excessively fast or slow degradation	Dual-network reinforcement, inorganic fillers, and scaffold-hydrogel composites	Adapt to bone defects of various sizes and morphologies	(Zhang Q. et al., 2023; Zhao et al., 2025)
Unclear long-term biosafety	Risks of drug residues, degradation products, and abnormal immune responses	Reduce effective doses and conduct long-term toxicological evaluations	Focus on ectopic osteogenesis, systemic toxicity, and immune responses	(Mndlovu et al., 2024)
Difficulties in clinical translation	Complex manufacturing, along with ununified evaluation standards and regulatory pathways	Standardized manufacturing, quality control, and large animal validation	Meet requirements for scalability, sterilizability, and regulation	(Foster et al., 2021; Qin et al., 2024)

Table 2 underscores that the clinical translation of dual-functional hydrogels cannot be achieved by optimizing material properties alone. Rather, it requires coordinated advances in release kinetics, antibacterial performance, regenerative microenvironment regulation, long-term biosafety, and standardized evaluation. The following sections discuss these key issues in greater detail.

### 6.1 Main scientific and technological challenges

The primary issue to resolve in the treatment of IBDs using loaded hydrogels is how to achieve the sequential and precise delivery of antibiotics and GFs (Rashidi et al., 2025; Rodrigues et al., 2025). An ideal scenario involves antibiotics rapidly establishing an effective local concentration in the early stage. Subsequently, GFs should be continuously released after the infection is controlled to support osteogenesis (Zhong et al., 2026). However, several challenges remain. For instance, the excessively rapid initial release of antibiotics can lead to local toxicity or premature depletion (Bardakova et al., 2026). Meanwhile, the biological activity of GFs is often compromised due to insufficient stability and overly rapid release (David et al., 2023; Ilhan et al., 2023). Researchers have begun utilizing dual-network or composite structural designs to optimize release kinetics. For example, a dual-network natural hydrogel (CMBG) was constructed through chlorogenic acid-magnesium ion coordination combined with genipin covalent crosslinking. This hydrogel can achieve the sustained release of antibiotics to a certain extent and effectively inhibit drug-resistant bacteria (Yao et al., 2022). Another hydrogel was constructed based on a poly(ethylene glycol)-poly(amino acid) block copolymer. After grafting BMP-2 peptides and introducing vancomycin, it could effectively coordinate early infection control with the subsequent bone regeneration process. Furthermore, this strategy showed effective infection control and bone regeneration in a rabbit radial model of IBDs (Yuan et al., 2022). Studies have also shown that loading vancomycin nanoparticles into a thermosensitive chitosan hydrogel system can provide sustained drug release for up to 14 days while promoting bone repair (Li J. et al., 2023). Although these strategies have shown encouraging preclinical results, a critical issue remains in current material design. It is necessary to make the drug release process more accurately match the actual clinical requirements of the various stages of infection control and tissue regeneration (Wu Y. et al., 2025).

Addressing complex infectious microbiomes is another significant challenge currently faced in this field. Clinical IBDs often involve polymicrobial infections, drug-resistant bacteria, or biofilm-associated infections. Single-antibiotic strategies are frequently insufficient to fully address these complex scenarios (Chen Z. et al., 2025; Qin et al., 2024). Consequently, a crucial direction in current research is the development of broad-spectrum or combinatorial antibacterial strategies. These approaches are often integrated with novel antibacterial technologies (Mutreja et al., 2022). For example, the CMBG hydrogel can simultaneously load vancomycin and polymyxin B. Combined with the synergistic effect of chlorogenic acid, it showed antibacterial activity against drug-resistant strains. These strains include methicillin-resistant *Staphylococcus aureus* (MRSA) and *E. coli* (Jiang et al., 2025). Phage-delivering hydrogels possess excellent biomolecule delivery capabilities. Therefore, they have been actively explored for the treatment of drug-resistant infections, such as orthopedic implant-associated bone infections (Kim et al., 2021; Yang X. et al., 2025). Furthermore, introducing antimicrobial peptides into self-assembling peptide hydrogel scaffolds is expected to provide a relatively sustained antibacterial effect for IBDs (Xu et al., 2024). Additionally, certain materials with inherent antibacterial activity have demonstrated promising application prospects. For instance, encapsulating single-layer Ti3C2Tx MXene and osteoactive carbon dots into a GelMA hydrogel can effectively promote the repair of IBDs (Xiao et al., 2025). However, more systematic research is still required to address several critical issues. These issues include the optimal combination methods for multiple antibacterial components, their specific release profiles, and long-term biosafety (Zheng et al., 2022).

In addition to anti-infection and osteopromotion, long-term biosafety and immunomodulatory effects are equally crucial. For implantable hydrogels, their degradation products and the residual local high concentrations of antibiotics and GFs can lead to severe consequences. These consequences include cytotoxicity, abnormal immune responses, or ectopic osteogenesis. Notably, the interaction between the material and immune cells, such as macrophages, inherently affects the local inflammatory microenvironment and the subsequent repair process. Studies have shown that an engineered *Bacillus subtilis* hydrogel loaded with sulfasalazine possesses excellent anti-MRSA infection properties (Fu et al., 2025). Furthermore, it can induce the polarization of macrophages toward the M2 phenotype. Consequently, this significantly enhances bone regeneration capacity (Chen C. Y. et al., 2025; Fu et al., 2025; Qiao et al., 2023). Hybrid hydrogels constructed from materials such as copper-doped bioactive glass can release ions, including *Cu* ^*2+*^, *Ca* ^*2+*^ and *Si* ^*2+*^, on demand. Thereby, they simultaneously participate in antibacterial action, immunomodulation, and bone remodeling. This effectively promotes infection control and bone tissue repair without the need for additional antibiotics or exogenous recombinant proteins (Huo et al., 2024; Jian et al., 2023; Kang et al., 2024; Lyu et al., 2025). Simultaneously, introducing silica nanoparticles loaded with vancomycin and insulin-like growth factor-1 (IGF-1) into a GelMA/poly(ethylene glycol) diacrylate (PEGDA) hydrogel results in a system with excellent biocompatibility. Moreover, this composite system can effectively promote the osteogenic differentiation of bone marrow stem cells (Kurian et al., 2023; Ren H. et al., 2025). Therefore, future research must not only focus on short-term antibacterial and osteogenic effects. It should also strengthen the comprehensive evaluation of systemic biocompatibility, metabolic processes, and long-term changes in the osteoimmune microenvironment.

### 6.2 Frontier trends and future perspectives

An important development direction for future research is the construction of smart hydrogels. These hydrogels should be capable of sensing changes in pH, enzymes, ROS, or temperature. Consequently, they can achieve more precise, on-demand drug delivery (Kumar et al., 2025; Wang X. et al., 2024; Zheng A. et al., 2023). For example, a pH-sensitive silk fibroin/sodium alginate hydrogel scaffold was developed. After loading with teicoplanin and aniline, it exhibits a faster drug release rate under alkaline pH conditions. This responsive release profile may allow the hydrogel to better adapt to dynamic changes in the local infection microenvironment (Motasadizadeh et al., 2022). Simultaneously, 3D bioprinting technology can be combined with patient imaging data, allowing the construction of scaffolds with morphologies and pore structures better matched to individual defect characteristics. This approach may facilitate more precise and personalized treatment strategies. A recent study combined a vancomycin-loaded hydrogel with a 3D-printed polycaprolactone scaffold loaded with FTY720, an angiogenic small-molecule drug. This bifunctional composite scaffold has been investigated as a potential platform for IBD repair (Baek et al., 2022). Smart hydrogels can also be further integrated with diagnostic functions. A recent study reported a structural color smart hydrogel patch (CPG) capable of monitoring local pH and temperature changes in real time and regulating drug release accordingly, suggesting its potential for integrated theranostic management of chronic osteomyelitis (Han et al., 2025). Combining such responsive functions with personalized scaffold design may further improve treatment precision in IBDs (Karthikeyan and Kang, 2025). Temperature-mediated antibacterial strategies, particularly NIR-triggered photothermal approaches, provide another route for externally controlled antimicrobial intervention. Their performance depends on careful control of thermal dose and tissue penetration, and recent analyses suggest that NIR-II may offer advantages over NIR-I for deeper infection sites because of its greater tissue penetration and imaging capability (Zhang et al., 2026; Zhang and Li, 2026).

In terms of development trends, hydrogel platforms are gradually transitioning from traditional bifunctional modes (antibacterial and osteogenic) to multi-modal systems. These advanced systems actively integrate immunomodulation, angiogenesis, and multi-signal delivery (Wang et al., 2025a). Existing studies demonstrate that the staged regulation of the immune microenvironment is often more conducive to improving the overall repair efficacy. For example, a sequential immunomodulatory hydrogel achieved the early release of *Ca* ^*2+*^. This was accomplished by introducing zinc-based nanoparticles doped with mineralized hydroxyapatite. This early release promotes M1 macrophage polarization and enhances infection inhibition. Subsequently, the sustained release of *Zn* ^*2+*^ guides the polarization of macrophages toward the M2 phenotype. This shift promotes osteogenic differentiation. Ultimately, it achieved effective remodeling of the osteoimmune microenvironment in an infected cranial defect model (Huang et al., 2025). Furthermore, the rational integration of angiogenic factors is equally crucial. One study incorporated VEGF, using sericin nanoparticles as carriers, into a sericin/poly(vinyl alcohol) (PVA) hydrogel system. The material properties were further improved by applying a gelatin coating. The results indicated that this system significantly promotes the proliferation of human umbilical vein endothelial cells (HUVECs). Furthermore, it upregulates the expression of angiogenesis-related genes, thereby enhancing its pro-angiogenic effects (Huang et al., 2025). Another study reported a conductive hydrogel based on copper nanoparticles. In addition to effectively eradicating bacteria, it can regulate macrophage phenotypic transformation via electrical signals. This regulation alleviates inflammatory responses and promotes angiogenesis. Ultimately, it facilitates the vascularized bone repair of IBDs (Qiu et al., 2025). Such multifunctional integration strategies may act at multiple stages of the repair process and thereby support more coordinated treatment of IBDs.

Pushing hydrogels loaded with antibiotics and GFs toward clinical application relies not only on improving the material properties themselves. It is also inseparable from the continuous optimization of clinical translation pathways. To achieve this, it remains necessary to establish more highly standardized animal models. These models must more accurately reflect the complex clinical conditions of patients. They are essential for conducting long-term safety and efficacy evaluations (Li Z. et al., 2023). Examples include large animal models or models accompanied by systemic diseases. More recently, a pH-responsive bisphosphonate hydrogel evaluated in an osteoporotic fracture-related infection model controlled MRSA while preserving cortical bone integrity by suppressing osteoclast activation and osteocyte apoptosis, further emphasizing the importance of testing multifunctional hydrogels under clinically relevant comorbid conditions (Li J. et al., 2026). For instance, researchers compared the therapeutic effects of antibiotic-loaded hydrogels and poly(methyl methacrylate) (PMMA) bone cement in a single-stage revision surgery. This was evaluated in an ovine model of chronic MRSA orthopedic implant-associated infection. Consequently, this study provided a crucial basis for assessing the clinical application potential of hydrogels (Foster et al., 2021). Simultaneously, it is necessary to further explore the feasibility of using hydrogels as delivery platforms for various bioactive molecules. These include small-molecule drugs, nucleic acids, and exosomes. This exploration will continuously expand their application scenarios (Ghosh et al., 2024). For example, one study combined rosmarinic acid, a natural product with both antibacterial and immunomodulatory activities, with chitosan to construct an injectable hydrogel. It was further applied in combination with tobramycin. This successfully formed a local microenvironment at the interface of the IBDs, simultaneously providing antibacterial and osteoimmune modulatory effects (Wang Z. et al., 2025). Another strategy involves combining antimicrobial peptides, the self-assembling peptide RADA16, and MSC-derived exosomes to construct self-assembling hydrogel scaffolds (Zhao et al., 2023). This system is used to simultaneously achieve sustained antibacterial effects and promote bone regeneration during the repair of IBDs. Additionally, incorporating bioactive components from traditional Chinese medicine (TCM) into hydrogel systems may provide another strategy for the treatment of bone infections through their multi-target effects (Jin et al., 2025). With the continued refinement of preclinical evaluation systems and the expansion of therapeutic payloads, the clinical potential of multifunctional hydrogels is expected to increase. However, therapeutic efficacy alone is insufficient to determine clinical feasibility. Translation of multicomponent hydrogel systems also requires reproducible manufacturing, sterilization, storage stability, quality control, and batch-to-batch consistency. These requirements become increasingly difficult to satisfy as hydrogels incorporate multiple drugs, nanoparticles, cells, or biologically active proteins. Changes in composition, cross-linking, sterilization, or storage conditions may alter degradation behavior and release kinetics and may also compromise the biological activity of GFs. Therefore, defining critical material and product attributes will be important for ensuring consistent therapeutic performance.

Clinical practicality should also be considered during material design. Parameters such as injectability, gelation time, defect geometry, and compatibility with surgical debridement and internal fixation may directly influence how these systems can be applied in patients. In addition, clinical IBDs vary substantially in infection severity, bacterial composition, defect size, vascular status, and host comorbidities, making it unlikely that a single hydrogel formulation will be suitable for all cases. Future development should therefore prioritize not only multifunctionality but also simplified formulation, scalable production, clinically practical administration, and standardized safety and efficacy assessment. For a complex indication such as IBDs, hydrogel systems with the greatest translational potential may ultimately be those that achieve an appropriate balance among antibacterial efficacy, immunomodulation, osteogenic activity, safety, and manufacturing controllability rather than those with the greatest functional complexity.

## 7 Conclusion

Bifunctional hydrogels loaded with antibiotics and GFs provide a novel approach for the treatment of IBDs. This approach is distinct from traditional staged treatments. Their core value lies in regulating infection control and bone regeneration through staged and hierarchical interventions. In the early stage, they inhibit bacteria and improve the inflammatory microenvironment. In the later stage, they continuously release osteogenic and angiogenic signals. Consequently, they achieve a more coherent repair process within a single integrated system.

Existing studies have demonstrated progress in material design, preclinical evaluation, and multifunctional integration. However, several barriers continue to limit clinical translation. Three priorities deserve particular attention: establishing reproducible and scalable manufacturing processes, defining long-term biosafety profiles, and developing standardized preclinical evaluation criteria that better predict clinical efficacy. Further optimization of stimuli-responsive delivery and coordinated regulation of immune, vascular, and osteogenic responses may improve therapeutic performance. Integration with technologies such as 3D bioprinting, stem cells, and exosomes may further expand the therapeutic scope of these systems. Addressing these material, biological, and translational issues will be important for advancing bifunctional hydrogels toward clinical application in infected bone defect repair (Mndlovu et al., 2024).
